# Dr. Hamilton’s Surgical Clinique, at Geneva Medical College

**Published:** 1847-02

**Authors:** 


### BUFFALO MEDICAL JOURNAL

AND

### MONTHLY REVIEW.

**VOL. 2.** FEBRUARY, 1847. **NO. 9.**

### ORIGINAL COMMUNICATIONS.

ART. I.—Dr. Hamilton's *Surgical Clinique, at Geneva Medical College. Reported for the Buffalo Med. Journal.*

*Ptosis.*—Dec. 3d 1846. Mrs. E. H. M., of Middlesex, Yates Co., aged 41. When eleven years old Mrs. M. had acute ophthalmia; from which time until the present the lids have been gradually falling; now the left eye is nearly closed, and the right partially. There is no appreciable thickening of the lids, nor are there granulations, to which the affection can be ascribed: but the patient is exceedingly nervous, and complains of great general muscular debility. The Ptosis may therefore be ascribed *in part* to an impairment of the levator from inflammation, but mainly to the exhausted condition of the whole nervous and muscular system. *Operation.* The integument of the upper lid of the left was seized with a pair of cross-bladed forceps, and cut away with scissors. The integument removed was oval shaped; in length corresponding with the length of the lid, and in breadth, six lines. The wound was closed with three sutures.

The sutures were removed on the third day. Patient returned home on the ninth day cured, so far as the eye is concerned.

*Staphyloma, Nebula and Chronic ophthalmia.*—Dec. 4th. Caroline B. coloured girl, aged nine years. Has had ophthalmia two years: right eye nebulous, staphylomatous and conjunctival vessels injected. Left eye suffering less. Patient feeble and emaciated; nearly blind

No operation at present required. Dr. H. recommends nit. arg. grs. ii to water ℥i as a collyrium: also plain, but nutritious diet, air, exercise, &c.; attention to the general health is regarded as most important.

*Strabismus internus, 3d degree.*—Dec. 3d. L. C. of Moravia; produced by whooping-cough when one year old; no other cases in family; has not changed its position much since first noticed; vision quite imperfect. Left eye.

*Operation.*—Patient seated upon a chair leaned his head against an assistant, who with his finger raised the lid. Dr, H. with the little finger of right hand depressed the lower lid, and directing the patient to evert the ball, he seized the conjunctiva with a pair of bull-dog-forceps and cut with straight, blunt pointed scissors. The tendon was then raised with a blunt hook and cut with the scissors. Three incisions were made with the scissors before the eye was entirely loosed. Eye became nearly straight: no apparent protrusion produced.

Patient recommended to keep eye cool with wet linen cloths for a few days — then exercise the eye by turning it out until the tenth or twelfth day, &c.

*Enlarged Tonsils.*—Dec. 4th. Infant child of ——— of Seneca Falls; aged 21 months. Glands enormously enlarged and have been so several months; the breathing very difficult; can be heard over the whole class room; is especially difficult at night; child often suffocates and becomes quite purple.

*Operation.*—The case was to difficult too be operated upon in the theatre. The next day Dr. H. reported that he had cut out one very thoroughly with Owen's instrument, but of the other he could only get about one-third. The child strangled and became quite purple during the operation ; the bleeding was insignificant — child now breathes freely.

*Tongue-tie.*—Dec. 7th. G. H. M. of Geneva, aged 5 years. Frænum linguæ extends far forwards; cannot speak distinctly; never had any difficulty in nursing.

*Operation.*—Cut the Frænum about three lines; but few drops of blood.

*Nævus*,—Dec. 8th. M. E. T. of Lima, Livingston Co. aged 12 years. The nævus was discovered when three weeks old on the under lip, and size of the head of a pin. Now size of a prune, oblong, extends well on inside of lip, of a deep purple color and growing rapidly.

*Operation.*—It was ligated by three strong ligatures; on the fourth day the face was considerably swollen and a slight hemorrhage occurred; the hemorrhage soon ceased and the swelling went down: on the sixth day she went home doing well.

*Necrosis of Humerus.*—Dec. 8th. C. A. P., aged 12 years, from Fayette; one year ago he was struck by a boy with the fist, near the upper end of his humerus; two weeks after a pain commenced at this spot, and in about two months it suppurated and opened, since which it has never closed: some fragments of bone have escaped. The head and shaft of humerus are both in a partly necrosed condition, a heavy shell having formed around the sequestrum; yet the sequestrum is not detached and it is not thought proper to enlarge the cloaca at present, for its removal. The health of patient is unimpaired: he is recommended to keep the sinus clean with castile soap and water and wait until the probe determines the sequestrum to be loose, when it should at once be removed.

Dr. H. stated that the case of necrosis operated upon at the close of the last term, H. K. aged 15, whose arm had been diseased seven years, was now well.

*Lipoma.*—Dec. 9th. Mrs. R. of Palmyra, aged 46 years, of full habit and accustomed to substantial diet, but temperate. Her nose became red and pimpled when 20 years old; this continued several years, but the integuments did not begin to thicken until after the application of a blister 12 years since.

It has been once removed by Dr. Mc Intyre, a surgeon of eminence at Palmyra, but it has regrown to more than its former size. The hypertrophy extends over half of the nose, including the alae, and gives to the organ a most unseemly bottle shape, the nose ending in an ugly tumor. Its color is dark red, which color extends broad upon the face: the sebaceous follicles are very large and constantly pour out upon the surface a very offensive matter; some of them are as large as a crow quill. Mrs. R. complains also of a sense of heat and weight in the nose and she suffers much from headaches, which she attributes to the state of her nose and face.

*Operation.*—All of the hypertrophied integuments were carefully dissected off from the end and alae of nose, leaving a very large raw surface to be slowly covered by the process of cicatrization. Dr. H. was very anxious to recover the wound by a plastic operation, taking the new skin, after the Tagliacozzian method, from the hand or arm, but she would not consent. The hemorrhage was less than was anticipated. A dressing of simple cerate was applied.

Six days afterward cicatrization had commenced.

*Chronic Pharyngitis,*—Dec. 10th. W. E. of Geneva, aged 23 This affection supervened upon a fever about six months since; patient feels a great constriction about the fauces and pharynx; has a constant acidity of stomach, and diarrhœa; looks feeble. A strong solution of nitrate of silver was applied to his throat, and directed to be repeated every other day. Internally he was to take a wine-glass full of lime water in a half-pint of boiled milk; diet chiefly animal: he must leave the store and take more exercise. Two weeks after patient had changed his employment and become a foot pedlar — his diarrhœa and acidity of stomach have ceased, and his throat is much improved.

*Chronic Conjunctivitis and Nebula.*—Dec. 11th. C. S. aged 21 years. Inflammation commenced one year since, acute and rapid, yet he was not bled from the arm — was cupped emeticised and catharticised. In four weeks they were nearly well; after two months became inflamed again, and thus they have continued to suffer under occasional attacks of subacute inflammation until the present. Right eye is now nebulous and vision is much impaired. He wears green glasses. Dr. H. directed him to throw away his glasses, bathe his eyes with cold water, walk out whenever the air was not too cold, eat plain but good food, use a green shade if the light hurt his eyes, &c. He also inserted a large seton in the back of his neck.

One week afterward his eyes had very much improved: in two weeks his right had mended one half. The seton discharges well and the result is ascribed mainly to this: all astringent collyria inflame his eyes and they are not therefore recommended.

*Nervous Deafness: hereditary.*—Miss. W. V. aged 22,—rather tall and thin, countenance pale, health tolerable but she is easily agitated. Three years since as she arose from the breakfast table in the morning she felt a roaring in the right ear and a deafness, both of which have existed more or less ever since. The left ear, also, at length is in the same condition. The deafness is not extreme, but it is worse when she is weary from sewing, or feels ill from any cause. Her mother has a similar deafness and roaring, which commenced at her 25th year and in the same way. She is also nervous and rather feeble.

The Eustachian tube is free, and the meatus externus.

She was advised to "go out to pasture"—ride on rough roads and in rough waggons, to use as a tonic the citrate of iron, and to stimulate the ear by dropping sweet oil and ol origanum in external ear. The prognosis is unfavorable.

*Amputation of finger.*—Mrs. D. of Geneva, aged about 40 During last summer, she had a thecal felon occuring on ring finger of right hand, the pus extending into the palm of the hand, and producing eventually a contraction of the flexor profundis of this finger. The second and third phalanx stood at right angles with the first and as she is a laboring woman, it was to her a great inconvenience.

She was advised by Dr. H. that the finger could be made straight by cutting the tendon, but that in the majority of cases where tenotomy had been practised upon the fingers the operation had resulted in a complete paralysis of the muscle cut. She preferred amputation.

*Operation.*— A single long flap was made on the palmar surface with a narrow scalpel and the amputation completed by disarticulation. No arteries required the ligature: three sutures and adhesive plaster were used and cold water dressings applied. The sutures were removed on the third day. It was entirely closed in a week: the inflammation had been very slight.

*Sarcomatous tumor.*—R. M. aged 26. Tumor under left angle of jaw, size of pullet's egg, oval, been growing four years, is now growing rapidly. He has another small tumor just above the clavicle, which commenced five months since. These tumors are enlarged Lymphatics, yet not scrophulous, not containing scrophulous matter, nor being liable to suppurate and open, but simple sarcomatous growths of the glands. They are much more amenable to therapeutic treatment than most other tumors, and much less to surgical, since if you cut out one, another is very likely to grow elsewhere. Patient is advised to take Tr. iod. gtta xv three times per day, and live on a very low, farinaceous diet, until his general plethora is reduced (he is now very plethoric) or until the tumors disappear: also to apply the iodine externally. If, however, the tumor grows in spite of all this, it should be cut out.

*Indolent Ulcer.*——— of Geneva. This lad now about 15 years old, had the right leg and part of the thigh terribly lacerated and almost deprived of its integument by a threshing machine, eight years ago. The wound has never closed entirely, but an indolent ulcer of great extent still exists, surrounded by a broad margin of hard integument from which sometimes new skin will form, and then it will rapidly crumble away, and the ulceration will extend, perhaps beyond its original bounds. Thus it has continued to partially close, and again open, during all this time; meanwhile the health and strength of the lad have remained excellent, but the leg has become bent at the knee and he walks with a halt. Two years ago Dr. H. took a cast of the ulcer, which is now seen to correspond almost precisely with its present extent.

Dr. H. and others having tried almost every mode of treatment which would offer a prospect of success, and having so completely failed, as Dr. H. believes because the indurated margin nearly two inches in breadth all around the sore is incapable of projecting from itself sound skin, the Dr. has proposed to the boy a *plastic* operation, with the view of planting upon the centre of the ulcer a piece of new and perfectly healthy skin. He proposes to take this from the calf of the other leg, (having secured the two together) not intending to cover over the whole sore, but perhaps two or three square inches, which he believes will be enough to secure the closure of the whole wound in a short time. Dr. H. claims the suggestion, whether it be regarded as good or bad, as his own, viz, the application of the plastic operation to old and indolent ulcers, yet he will postpone getting it patented, until he learns how his Boston friends get along with their ether patent.

*Indirect Inguinal Hernia, Congenital.*—Infant son of C. Y. of Rose, Wayne Co. aged 14 months. The hernia, (left side) was noticed next day after birth: it has been constantly down, and is now quite large. A German doctor of Lyons has been applying an ointment which he said would cure it! but it made the parts quite sore and the parents discontinued it. Child has never worn a truss. Dr. H. applied a small Hull's truss which promises to keep it up very well.

*Enlarged Tonsils.*—M. A. aged 16, from Phelps. They began to enlarge three years since, has had repeated attacks of tonsillitis, especially during the winter. They were not very large, and Dr. H. removed only a portion of the largest, with Owen's tonsil instrument. Scarcely any hemorrhage.

*Deafness.*—H. T. aged 6 years, from Naples. Had from infancy, until within the last year or two, occasional pain and suppuration in ears; discharge was very offensive. At present can hear only the loudest sounds; cannot articulate but a few words; looks intelligent; considerable wax in both ears.

Here is a destruction of the structure of the drum and ossiculi from inflammation and suppuration: this has now ceased, and the treatment is directed simply to cleansing out the external ear, and preventing a re-accumulation of the cerumen in such excess. The ears are to be carefully syringed with castile soap and warm water, and when clean a drop of the following is to be let into the ear twice per day; ol. oliv. ℥iv nit. merc. ung-℥i.

*Strabismus Convergens.*—Miss. E. M. aged 12 years, from Phelps. Has strabismus of left eye; it commenced when she was two years old without any apparent cause; the squint eye is short-sighted. Dr. H. refused to operate because the eyes were prominent, and if a very slight projection of the ball should follow the section of the muscle it would be a more serious deformity than the squint, and because also the eye was turned but slightly — first degree — and the operation might produce a strabismus divergens.

*Enlarged Tonsils.*—Miss. H. C. aged 22. Miss. C. had measles three years since which was succeeded by enlargement of both tonsils. They are now about the size of a filbert; they inflame occasionally, but rarely become much larger, yet they are almost constantly painful, and the pain is especially intense just preceding menstruation. It was this last circumstance, the pain, which induced Dr. H. to cut them off. They were both *thoroughly* removed—hemmorrhage very slight.

*Membranous Pterygeum.*—H. P. T. aged 47, from Benton, Yates Co. Patient had acute opthalmia about 25 years since, from which time a pterygeum has been growing from inner canthus of the right eye; it has now encroached two lines upon the cornea, and interferes considerably with vision.

*Operation.*—The whole breadth of the pterygeum was seized with a pair of bull-dog forceps and cut away with the scissors, the conjunctiva not being disturbed anterior to the junction of cornea and sclerotica, nor farther in the opposite direction than within two lines of the caruncle, lest, from a too extensive dissection in this latter direction, a squint should be produced by the contraction of the cicatrix. Vessels were now seen coursing in the cellular texture, and they were removed by a second incision. The patient was directed to live low, and keep the eye cool with linen cloths wet in cool water, &c.

Dr. H. did not, as he generally does not in these cases, promise that the pterygeum will not return.

The report of the clinique will be continued through the term.

P *****, *Reporter for the Journal.*

